# P-719. Respiratory syncytial virus (RSV) disease burden in older adults with acute respiratory infection (ARI) in Europe during the 2022/2023 RSV season

**DOI:** 10.1093/ofid/ofae631.915

**Published:** 2025-01-29

**Authors:** Rosa Prato, Marek Mital, Sílvia Narejos Pérez, Georg-Eike Böhme, Ronald-Paúl Torres Gutiérrez, Charles Bundy, Susannah Eyre, Philip Joosten, Theo Last, Shubhangi Gawade, Jean-Yves Pirçon, Veronica Hulstrøm, Pouya Saeedi

**Affiliations:** University of Foggia, Foggia, Abruzzi, Italy; Clinical Agnieszka Mital Centrum Badan Clinic, Elblag, Poland, Warsaw, Mazowieckie, Poland; CAP Centelles, Barcelona, Catalonia, Spain; Medical Center Motorworld, Munich, Bayern, Germany; Hospital Nuestra Señora de Sonsoles (CAAV), Ávila, Castilla y Leon, Spain; Trowbridge Health Centre, Trowbridge, England, United Kingdom; Elpida Clinical Trials, Wallasey, England, United Kingdom; GSK, Wavre, Brabant Wallon, Belgium; GSK, Wavre, Brabant Wallon, Belgium; GSK, Wavre, Brabant Wallon, Belgium; GSK, Wavre, Brabant Wallon, Belgium; GSK, Wavre, Belgium, Wavre, Brabant Wallon, Belgium; GSK, Wavre, Brabant Wallon, Belgium

## Abstract

**Background:**

RSV is a highly contagious pathogen that can cause severe disease in older adults. We assessed the RSV disease burden among older adults.

**Figure:**
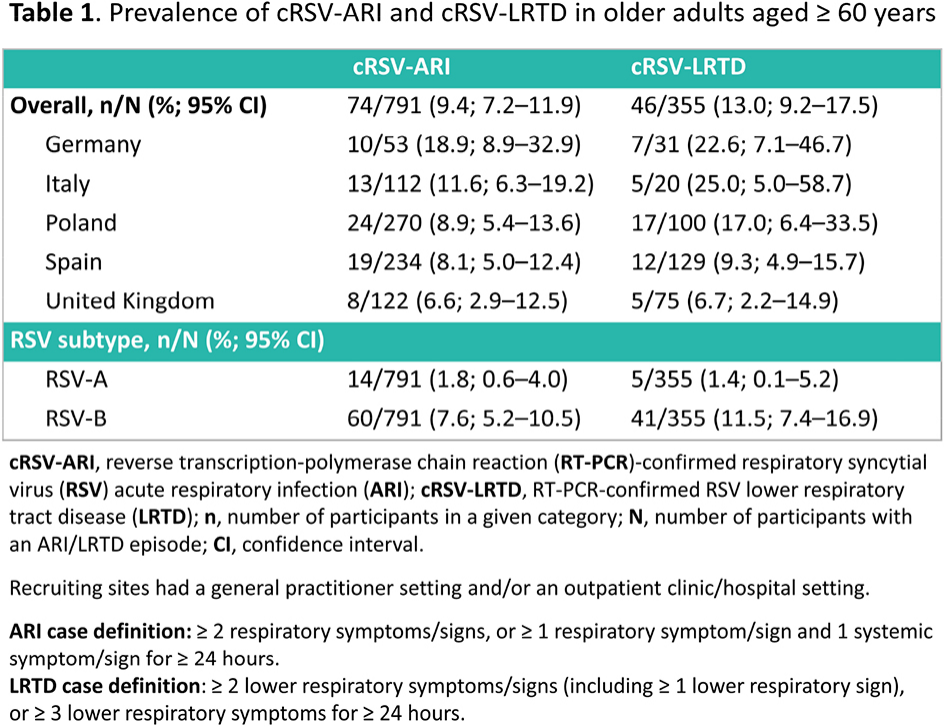

**Methods:**

We present results from the second RSV season (1-Oct-2022/30-Apr-2023) of this ongoing prospective epidemiological study covering 3 consecutive RSV seasons in which adults aged ≥ 60 years from 5 European countries were followed up for ARI symptoms 28 or 56 days if ARI was not resolved by day (D)29. Medical conditions, nasal and throat swab samples for RT-PCR detection of RSV and other respiratory viruses were collected at D1.

**Figure:**
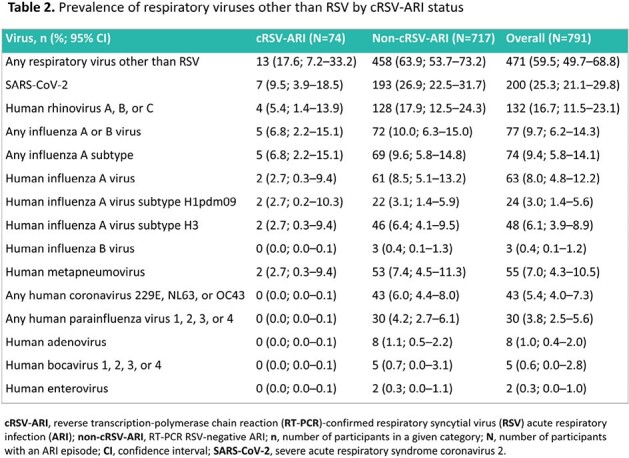

**Results:**

Of 791 participants with an ARI episode, 74 (9.4%) had RT-PCR-confirmed RSV-ARI (cRSV-ARI, 60–74-year-olds: 53/626 [8.5%]; ≥ 75-year-olds: 21/165 [12.7%]) with variation between countries (**Table 1**). Median duration of the ARI episode was 21 days (range: 5–59) for cRSV-ARI versus 19 days (range: 4–66) for non-cRSV-ARI. Of participants with cRSV-ARI, 70/74 (94.6%) had both upper and lower respiratory symptoms, and 46/74 (62.2%) met the lower respiratory tract disease (LRTD) case definition (**Table 1**). The most frequently recorded upper respiratory symptoms, lower respiratory symptoms, and systemic symptoms were nasal congestion/rhinorrhoea (63/74 [85.1%]), cough (72/74 [97.3%]), and fatigue (47/74 [63.5%]), respectively. RSV activity peaked in December and January (**Figure 1**). 68/74 (91.9%) participants with cRSV-ARI and 43/46 (93.5%) participants with cRSV-LRTD had ≥ 1 current and chronic medical condition. The most frequent chronic medical conditions were vascular (cRSV-ARI: 48/74 [64.9%]; cRSV-LRTD: 30/46 [65.2%]) and metabolic disorders (cRSV-ARI: 47/74 [63.5%]; cRSV-LRTD: 29/46 [63.0%]). The most common respiratory viruses other than RSV were SARS-CoV-2 and rhinovirus (**Table 2**).

**Figure:**
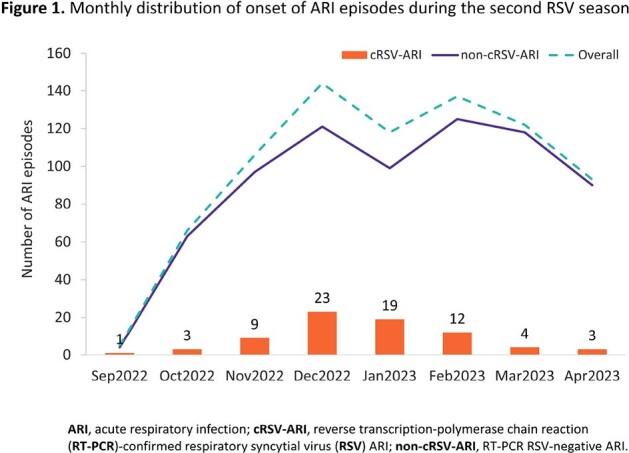

**Conclusion:**

While RSV activity shifted during the COVID-19 pandemic, RSV seasonality during the 2022/2023 season tended to return towards pre-pandemic trends in Europe, with peak activity during the winter months. Country-level prevalence varied, with higher prevalence in Germany and Italy vs other countries. However, the overall prevalence in outpatient settings was within the range of pre-pandemic data from a recent meta-analysis. Future data will enhance understanding of post-COVID RSV epidemiology.

**Funding:** GSK

**Disclosures:**

**Rosa Prato, M.D.**, Merck & Co., Inc.: Advisor/Consultant|Merck & Co., Inc.: Grant/Research Support **Philip Joosten, PhD.**, GSK: Employment|GSK: Stocks/Bonds (Public Company) **Theo Last, Pharm.D**, GSK: Employment|GSK: Stocks/Bonds (Public Company) **Shubhangi Gawade, MSc.**, GSK: Employment **Jean-Yves Pirçon, PhD.**, GSK: Employment|GSK: Stocks/Bonds (Public Company) **Veronica Hulstrøm, MD, PhD**, GSK: Salary as GSK employee with stock options|GSK: Stocks/Bonds (Public Company) **Pouya Saeedi, PhD.**, GSK: Employment

